# Supplementary material for: Are carbon emissions trading and green financial instruments synergistic? -Comprehensive quantitative research based on content analysis
Source: PLoS One. 2024 Mar 7;19(3):e0298601. doi: 10.1371/journal.pone.0298601 (PMC10919601; doi:10.1371/journal.pone.0298601)
Supplement: S1 Data — (ZIP) [file pone.0298601.s001.zip › Data/地级市Z_F工作报告.zip!/╣π╢1⁄2/╤⌠╜¡╩╨/└┤╫╘╣1⁄2╓┌║┼í╛┬φ┐╦╩2╛▌═°í┐_2006.docx]

各位代表：

　　我代表市人民政府向大会作政府工作报告，请予审议，并请政协各位委员和其他列席人员提出意见。

　　　　　　　　　　　　　　　　“十五”时期工作回顾

　　“十五”时期是我市经济社会发展取得较大成就的五年。五年来，在省委、省政府和市委的正确领导下，在市人大及其常委会和市政协的监督支持下，市政府坚持以邓小平理论和“三个代表”重要思想为指导，深入贯彻中共十六大及各次全会和中央政治局委员、广东省委书记张德江及省长黄华华视察阳江重要讲话精神，认真落实科学发展观和构建社会主义和谐社会的战略部署，结合阳江的实际，进一步完善发展思路，狠抓发展第一要务，在全市人民的共同努力下，国民经济平稳较快健康协调发展，社会各项事业全面进步，精神文明建设和民主法制建设取得新成绩，“十五”计划的各项目标任务全面完成。

　　——经济综合实力跃上新台阶

　　2005年，全市生产总值完成295亿元（按原核算方法为318.5亿元），比上年增长14.3%；人均11801元，比上年增长14%，增速均创近十年新高。生产总值和人均生产总值五年年均分别增长11.8%和11.2%。2005年地方财政一般预算收入8.7亿元，比上年增长17.2%，五年年均增长20.7%。全市金融机构人民币各项存款余额245.4亿元，比2000年增长76%，贷款余额99.7亿元，剔除剥离贷款后比2000年增长16.9%。贯彻落实中央宏观调控政策取得初步成效，产业结构进一步调整优化，三大产业比例由2000年的39.8∶29.5∶30.7调整为2005年的30.4∶36.6∶33，第二、三产业比重2004年起均超过第一产业。

　　工业经济快速发展。2005年实现工业总产值328亿元，比上年增长18%，五年年均增长18%。工业产业结构进一步优化升级。在规模以上工业中，钢铁、石化、汽配等重工业所占比例由2000年的24.8%提高到31.9%。电力能源工业取得新突破。阳江核电站、阳西燃煤电厂建设进展顺利，阳江抽水蓄能电站和海陵岛风力发电场的前期工作有序推进。临港工业起步良好。引进了同济大学天虹（阳江）科技园等重点企业，上马建设了广东丰源粮油临港加工区等一批重点工业项目。高新技术产业有新发展。建立了省级阳江高新技术产业开发区和阳东民营科技园，培育发展了一批高新技术产业和民营科技企业，2005年高新技术产业实现产值41亿元，比上年增长38.9%，五年年均增长34.6%。传统产业发展水平不断提升。形成了五金刀剪、轻纺服装、机电建材、食品加工、医药化工和家具编织等支柱产业为主的工业体系。其中五金刀剪行业发展迅猛，2005年实现产值80亿元，是2000年的2.4倍，五年年均增长19.1%。中国菜刀中心、剪刀中心、小刀中心先后落户我市，并荣获“中国刀剪之都”和“中国五金制品（刀剪工具）出口基地”称号，阳东县被评为省产业集群升级示范区。成功举办了四届国际刀剪博览会，五金刀剪品牌进一步提升。名牌带动战略成效显著。“十五”期间，新增中国驰名商标1件、中国名牌产品1个、国家免检产品4个，原产地域保护产品2个、商务部重点培育和发展的出口品牌1个、广东省著名商标23件、广东省名牌产品14个。

　　农业经济稳步发展。2005年实现农业总产值139.8亿元，比上年增长6.5%，五年年均增长6.4%。粮食生产得到加强。2005年粮食种植面积229.2万亩，比上年增加1.2万亩；粮食总产量76.7万吨，比上年增长1%。农业结构不断优化。效益农业和特色农业迅速发展。花卉、蚕桑等经济作物种植面积进一步扩大。粮经比例由2000年的48.2∶51.8 调整为2005年的44.5∶55.5。农业产业化进程加快。至2005年底止，培育了省级农业龙头企业4家、市级农业龙头企业10家、农业专业化合作组织108个，带动农户25.4万户，占农户总数的54.3%，户均增收1055.7元。农田水利基本建设不断加强，小型水库基本实现除险加固达标。林业、畜牧业进一步发展，海洋经济不断壮大。高致病性禽流感和重大动物疫病得到有效防控。

　　第三产业较快发展。2005年社会消费品零售总额158.9亿元，比上年增长15.3%，五年年均增长12.9%。“十五”期间，连锁经营店、仓储式商场、超市等商业网点日益增多。文化、体育、餐饮、医疗保健等服务业稳步发展，汽车、通讯、住房等消费需求畅旺。2005年商品房销售面积64.5万平方米，销售额9.3 亿元，分别增长33.2%和51.3%。

　　——招商引资和对外贸易取得新成效

　　投资软环境进一步优化。招商引资政策不断完善，出台了《阳江市进一步加强招商引资工作的决定》、《阳江市招商引资奖励办法》、《阳江市降低收费门槛，促进招商引资的决定》等文件，利益分享政策和奖励措施进一步落实。清费减负工作全面开展，2004年以来共取消或减少行政性收费41项。机关作风建设不断加强，服务水平和办事效率有较大提高。招商引资责任制有效落实。制定实施了《阳江市招商引资和发展工业目标考核奖惩办法（试行）》，各级招商引资的主动性和积极性日益提高。招商引资势头良好。与发达地区共建产业转移园区取得新突破，中山火炬（阳西）、中山石岐（阳江）两个产业转移工业园被批准为省级产业转移工业园。大企业、大项目引进步伐加快，中国广东核电集团、广东珠江投资有限公司、中国保利集团、广东粤电集团、澳门吴福集团、日本山光社等企业先后投资阳江。2005年实际利用外商直接投资0.76亿美元（新口径），比上年增长31.3%。外贸进出口稳定增长。2005年完成进出口总额10.3亿美元，五年年均增长10.6%；其中外贸出口9.1亿美元，五年年均增长11%。全方位开放格局逐步形成。与117个国家和地区建立了经贸关系，出口商品达400多种(类)。特别是与东盟、北美和港澳地区的经贸往来日益密切，对外经济合作与交流进一步加强。

　　——区域特色经济实现新发展

　　民营经济发展迅猛。“十五”期间新增民营工业企业1170家，新增产值127.4亿元。2005年民营工业完成产值219.4亿元，增长16.2%，占全市工业总产值66.9%。旅游业稳步发展。2005年接待游客421.1万人次，比上年增长14.8%，五年年均增长19.1%；旅游总收入21.6亿元，比上年增长22.7%，五年年均增长17.9%。景区景点开发建设不断完善，阳江温泉度假村和海陵岛东方银滩欢乐广场等一批旅游项目竣工投入使用，阳西月亮湾健康旅游度假村、阳江涛景度假村、海陵三山岛国际旅游度假区等旅游项目建设加快。连续举办五届旅游文化节，旅游形象有效提升，阳江被评为中国优秀旅游城市，阳春凌霄岩被评为国家地质公园，海陵岛被评为全国十大最美海岛之一。海洋渔业发展加快。2005年水产品总量91万吨，总量和人均占有量连续7年居全省第一。渔业总产值51亿元，比上年增长8%，五年年均增长7.2%。海洋捕捞、海水养殖和水产品加工与流通全面发展。国家级标准化吊桩牡蛎和泥蚶养殖基地、广东省重要对虾生产基地相继建成。渔港建设不断加强，闸坡渔港被评为国家级中心渔港，东平、沙扒渔港被评为全国一级渔港。成功举办了三届南海（阳江）开渔节，打响了海洋渔业品牌。南药生产加工业发展势头良好。2005年南药种植面积7.9万亩，产值2.3亿元，分别比上年增长26.6%和29.1%。春砂仁、穿心莲、板兰根、霍香等南药的种植规模不断扩大，形成了一批专业性较强的中药材种植园、良种示范园。同时，引进了一片天医药集团等医药企业进行南药深加工。

　　——基础设施和环境建设取得新突破

　　“十五”时期，全市完成固定资产投资293.6亿元，五年年均增长11.3%。其中，2005年完成80.4亿元，比上年增长41.7%。交通、能源、通信等基础建设成效显著。“十五”期间全市交通基础设施建设累计完成投资45.6亿元，比“九五”期间增加10.3亿元，广东西部沿海高速、开阳高速、阳茂高速、省道113线新兴至潭水段等公路建成通车，阳江至云浮高速公路建设前期工作有序推进，实现了市至省城通高速公路，市至县通一级公路，县至镇通二级公路，镇至行政村通机动车。2005年底，全市公路通车里程4836公里，公路密度62公里/百平方公里。阳阳铁路全线拉通运营。阳江港5万吨级航道疏浚工程顺利完成，3万吨级粮食码头建成投入使用，“十五”期间港口吞吐量比“九五”期间提高565.9万吨。城乡电网建设进一步完善，完成了220千伏阳江变电站扩容工程，新建或扩建了桂山、永宁、闸坡等11个110千伏输变电工程。全市通信光缆总长4万芯长公里，信息化指数48%。城市建设日新月异。新汽车总站、中国刀具城会展中心、文化广场、青少年宫、垃圾滤液处理厂和医疗废物处理中心等一批市政工程和公共设施竣工投入使用。西平路、新江北路、体育路、安宁路等20多条城市主干道建成开通，人民广场、鸳鸯湖广场等公众活动场所得到改造建设，城南新区的规划建设已经启动。一批新型公寓式住宅小区相继建成，城市环境和面貌发生深刻变化，被评为“广东省卫生城市”。市区建成区面积由2000年的24.5平方公里扩展到35.3平方公里。阳春、阳东和阳西城区不断拓展，中心镇建设加快，城镇化水平进一步提高。

　　——体制改革迈出新步伐

　　国企改革稳步推进。潭水水泥厂、八甲电厂等一批国有企业改制工作顺利完成，引进了粤电集团参与阳江港开发总公司的改制。行政管理体制不断完善。市县镇三级政府机构改革和撤并镇工作有效推进，市级行政编制精简32%，全市撤并了9个镇。行政审批制度改革取得初步成效，取消行政审批事项56项。农村税费改革取得阶段性成果。2003年以来，共免征农业税费4亿元，农民人均减负227元。农村信用社试点改革顺利推进。粮食流通、投融资、科技、文化、卫生等体制改革不断深化，体制性障碍进一步消除。

　　——文化名城建设取得新进展

　　教育事业较快发展。“十五”时期投入教育建设资金3.8亿元，新建校舍55.1万平方米，改造薄弱学校293所、老区危房学校159所，18所中小学被评为省一级学校。“普九”教育整体水平不断提高，“以县为主”的基础教育管理体制进一步完善，义务教育“一费制”全面实施。减免农村贫困家庭子女义务教育阶段书杂费工作有效落实，免收了32467名贫困学生的书杂费。高中阶段教育加快发展，2005年全市普通高中在校生人数41448人，比2000年增长101.6%。由市政协倡议社会各界捐资建设的同心中学已于2005年秋季招生，引进民资兴建的阳东广雅中学建设加快，阳春一中、二中的迁扩建工作顺利进行。高等教育实现新突破，成立了阳江职业技术学院，办学规模逐年扩大，在校生达6323人。职业教育和成人教育不断发展。教育信息化取得新成绩。科技工作不断加强。累计实施国家和省级科技计划项目119项、“科技兴市”工程等项目85项，分别培育了1家国家级和55家省级高新技术企业以及127家省级民营科技企业。以企业为主体的科技创新体系不断完善，扶持企业组建了6家省级工程技术研发中心和2家省级农业科技创新中心，培育了2个省级和9个市级专业镇技术创新试点，建立了市五金刀剪技术创新中心。科研水平日益提高，“十五”期间，共完成科研项目115项，其中3项获省科技进步奖；产学研合作不断加强，建立了华南理工大学阳江工业技术研究院，举办了3届科技成果推介洽谈会。知识产权工作取得新成绩，5年专利申请量达2126件，保持粤西地区领先地位。人才战略稳步实施。粤西区域性人才市场加紧筹建，人才交流服务不断完善，人力资源管理逐步加强。“百万农村青年技能培训工程”和创业培训工程扎实推进，与阳江经济发展相适应的各类人才不断增多。文化事业和文化产业蓬勃发展。文化设施建设进一步完善，广东海上丝绸之路博物馆正式动工，市图书馆、科技馆、博物馆和群众艺术馆等文化设施加快建设。文化管理体制改革有序推进，组建了文化广电新闻出版局。群众文化娱乐活动丰富多彩，文化创作日趋活跃，《漠海钩沉》荣获全国“山花奖”。阳江被授予“中国诗词之市”和“中国楹联文化城市”称号。

　　——精神文明和民主法制建设取得新成绩

　　公民思想道德建设不断加强。深入开展“爱国、守法、诚信、知礼” 为主要内容的现代公民教育活动，公民素质和城市文明程度不断提高。群众性精神文明创建活动深入开展，连续两次被授予“广东省文明城市”称号。民主法制建设进一步加快。坚持向人大及其常委会报告工作、向政协通报情况，认真执行人大及其常委会的决议，自觉接受人大及其常委会和政协的监督。“十五”期间，市政府办理人大议案10件、代表建议454件、政协建议案7件、政协委员提案432件，办复率100%。认真听取各民主党派、工商联、无党派人士和专家学者对政府工作的意见，与工会、共青团、妇联等人民团体的联系更加密切。“四五”普法规划圆满完成，公民法律素质普遍提高，法律服务体系日益完善，对特殊弱势群体的法律援助深入开展。基层民主建设不断加强，村委会换届选举工作顺利完成，政务、厂务、村务公开和农村居民自治等制度逐步完善，阳东县北惯镇平地村被授予“全国民主法治示范村”称号。依法行政工作全面推进，行政执法和廉政建设责任制有效落实，公务员队伍依法行政能力和效率有了新的提高。

　　——和谐阳江建设呈现新面貌

　　社会保障进一步加强。就业和再就业工作取得新成绩，“十五”期间新增就业岗位9万个，安置下岗失业人员再就业2.3万人，其中“4050”人员6000人，城镇登记失业率控制在3.5%以下。社会保险覆盖面不断扩大，参加养老、失业、医疗、工伤、生育保险的人数逐年增加。城乡居民最低生活保障制度和农村五保供养制度不断完善，至2005年底，共有88492名城乡居民获得最低生活保障，8434人落实五保待遇。

　　人民生活水平日益提高。2005年全市在岗职工年平均工资13640元，农民年人均纯收入4368元，五年年均增长10.8%和4.8%。城乡居民储蓄存款余额189.9亿元，比2000年增加84.9亿元，年均增长12.6%。“十项民心工程”稳步推进，特别是农村饮水工程成效明显，解决了32.8万人的饮水难问题。群众住房难、就医难、打官司难和子女入学难等问题逐步缓解。

　　社会保持和谐稳定。社会治安综合治理不断加强，防控体系进一步完善， “双抢”、“两盗”等犯罪活动受到严厉打击， “六合彩”赌博和“黄赌毒”等社会丑恶现象得到有效遏制，社会治安状况不断好转。安全生产管理进一步加强，横水渡船、码头改造、烟花爆竹厂转产等专项整治工作成效明显，安全生产形势稳定。消防工作成绩突出，市政府被授予“2002-2005年度全省消防工作先进单位”荣誉称号。严厉打击非法传销、制假售假等违法活动，市场经济秩序明显好转。做好群众来信来访工作，认真查处农村征地、城镇房屋拆迁、国企改革等工作中损害群众利益的行为，积极清理建设领域拖欠工程款和农民工工资，预防和妥善处置群体性事件，社会和谐稳定。

　　社会各项事业不断发展。全民健身运动深入开展，竞技体育水平不断提高。市体育中心一期工程体育馆建成使用。成功承办了九运会沙滩排球赛和男子排球赛、全国热气球锦标赛、世界华人风筝赛暨第六届中国（阳江）国际风筝会等大型体育赛事。城乡爱国卫生、医疗服务、疾病控制和药品监督工作全面加强。公共卫生体系建设进一步加快，新建了市疾病防控中心大楼、市人民医院感染性疾病中心大楼。突发公共卫生事件预警和应急机制日益完善。无偿献血工作成绩显著，连续三次荣获“全国无偿献血先进城市奖”。人口与计划生育工作成绩突出。在全国率先实行“节育奖”，计生依法行政、综合治理、宣传教育、信息化管理等工作经验在全省推广，人口自然增长率由2000年的6.76‰降至2005年的6.51‰，连续8年获得省政府表彰。国土资源保护工作取得新成效，连续5年实现耕地占补平衡。环境生态建设得到加强。烟尘控制区、环境噪声达标区覆盖率分别达100%和85.2%，市区绿化覆盖率26.3%，全市森林覆盖率53.1%，城乡居民生活环境和质量明显改善。环境保护责任制考核2003年和2004年连续两年被省评为优秀。国防动员、民兵预备役和双拥优抚工作成效显著，连续4次被评为“全省双拥模范城”，2003年被评为“全国双拥模范城”。防震减灾工作取得新进步，2005年荣获全国防震减灾工作优秀奖和地震监测预报单项奖以及全省市、县防震减灾工作第一名。档案服务社会功能逐步增强。民族、宗教、外事、侨务、统计、妇女儿童、老龄、人防、打私、气象、广播电视、新闻出版、社会科学、市志、残疾人工作等各项社会事业均取得较大成绩。

　　各位代表！“十五”时期，我市经济社会发展取得了较大的成绩，为今后的发展奠定了良好基础。这是省委、省政府和市委正确领导的结果，是各级人民代表大会及其常委会和人民政协监督支持的结果，是全市人民团结一致，开拓进取，心齐实干的结果。在此，我代表市人民政府向全市广大工人、农民、知识分子、干部职工、驻阳江人民解放军、武警部队官兵和公安干警以及各界人士致以崇高的敬意！向港澳台同胞、海外侨胞和关心支持阳江建设的国际友人表示衷心的感谢！

　　回顾五年来的工作，我们深深体会到，实现阳江加快发展、跨越发展、协调发展和可持续发展，要切实做到“六个必须”：一是必须增强责任意识、危机意识、机遇意识和发展意识，抢抓机遇加快发展；二是必须树立和落实科学发展观，促进经济发展和社会全面进步；三是必须立足当地实际，不断完善发展思路；四是必须优化发展环境，增创发展新优势；五是必须通过重点突破，以点带面推动全局；六是必须坚持执政为民，切实维护群众利益。

　　我们也清醒地看到，我市经济社会发展中还存在不少困难和问题，主要是：经济总量小，结构不合理，产业竞争力不强；固定资产投资不足，招商引资规模小，短期内对经济发展拉动作用不明显；税源不足，财政收支平衡压力较大，城乡建设和社会事业欠帐较多；科技创新能力较弱，教育质量有待提高，高素质人才缺乏；劳动就业压力加大，社会保障体系仍需完善，部分群众生产生活仍然困难；政府职能和机关作风转变未完全到位，社会治安治理任务繁重，投资环境有待进一步改善等。对此，我们将采取切实措施认真加以克服和解决。

　　　　　　　　　　　“十一五”时期的奋斗目标和主要任务

　　“十一五”时期，是我市追赶全省发展步伐，实现与全省同步全面建成小康社会、率先基本实现现代化的关键时期。我市的发展既面临难得的机遇，又面临严峻的挑战，但总体上机遇大于挑战。从内部形势看，我市有18年经济发展的良好基础；有日臻完善的基础设施和愈发凸显的区位优势；有重大项目的强力拉动；有丰富的自然和人力资源；有承接发达地区产业转移的良好机遇。尤为重要的是，经过多年的探索，我市发展观念和发展思路不断创新完善，全市上下形成了加快发展、科学发展的共识，特别是市第四次党代会后，我市提出重点发展临港工业和区域特色经济，打造广东电力能源基地、建设面向西南的物流配送基地的发展思路，对于我市加快发展、全面建设小康社会具有长远的战略指导意义。从外部形势看，世界经济继续保持增长，结构调整加快。国内政治社会稳定，经济平稳较快发展。省委、省政府加大对东西两翼和山区的支持力度，CEPA的实施和“泛珠三角”区域合作战略的推进，有利于我市发挥错位竞争优势，加快发展。但是，我们必须看到，我市经济社会发展中还存在许多不容忽视的困难和问题，特别是国际形势错综复杂，国内省内改革开放不断深化，部分同类地区迅速崛起，进一步增大了我市加快发展的压力。因此，我们必须把加快发展作为主题，进一步转变发展观念，创新发展模式，增强发展动力，加快发展速度，提高发展质量，力争“十一五”期间经济社会发展实现新的突破。

　　根据党的十六届五中全会、中央经济工作会议、省委九届七次、八次全会精神和市委关于“十一五”规划的建议，市政府组织编制了《阳江市国民经济和社会发展第十一个五年规划纲要（草案）》。“十一五”时期我市经济社会发展的奋斗目标是：经济综合实力明显增强，产业结构明显优化，社会事业明显进步，城乡居民收入水平和生活质量明显提高，社会服务和管理明显改善，民主法制建设更加健全，人口、资源、环境更加协调，社会更加和谐。经济发展的主要预期目标是：生产总值年均增长12%以上，单位生产总值能耗年均下降2.8%，工业总产值年均增长20%，全社会固定资产投资年均增长15%，社会消费品零售总额年均增长13%，实际利用外商直接投资年均增长25%，出口总额年均增长10%，地方财政一般预算收入年均增长10%。

　　做好“十一五”工作，实现“十一五”发展目标，必须在“六个着力”上下功夫：

　　一是着力提高经济发展速度。坚持发展为第一要务，抢抓机遇，乘势而上，加快经济发展速度，尽快缩短与发达地区的差距。重点抓好电力能源、临港重化工业、轻工制造业、物流配送、特色农畜产品生产、海洋产业、中药材生产加工、文化生态休闲旅游等八大基地建设，打造经济亮点，培育经济引擎，加快经济发展速度，提高经济增长的质量和效益，做大经济总量。

　　二是着力抓好招商引资。把招商引资作为加快发展的突破口，加大园区载体建设力度，以引进大项目为重点，进一步落实招商引资责任制，强化招商引资实绩考核，加强招商引资项目的跟踪落实，努力引进一批上规模、对地方财税贡献大的大企业大项目，提高招商引资规模和水平，实现以大开放、大引进促进大发展。

　　三是着力营造区域竞争优势。继续加强交通基础设施建设，形成更为完善的综合交通运输网络，强化区位交通优势。加大旅游、海洋、温泉、南药等自然资源的开发力度，充分利用好资源优势。加快推进能源项目建设，增创能源优势。发展和巩固五金刀剪等传统优势行业，培育提升特色产业优势。大力优化发展环境，营造服务优势，形成与珠三角及相邻地区的优势互补和差异竞争优势。

　　四是着力促进城乡区域协调发展。加大工业带动农业的力度，推进农业产业化、集约化、效益化；加大城市“反哺”农村力度，充分发挥城市和中心镇的辐射带动作用，推动农村经济发展。加大县级经济发展的协调力度，引导各地根据自身条件选准重点，各展所长，防止低水平重复建设，促进各县(市、区)强化优势、互动发展。

　　五是着力提高自主创新能力。全面实施科技创新“五大工程”，加快建立完善以企业为主体的技术创新体系，提高企业自主创新能力。积极运用高新技术改造传统产业，培育和发展高新技术产业，提升产业整体竞争力。加强产学研合作，推动科研成果转化。加强知识产权工作，培育和发展一批知识产权优势企业。大力发展信息产业，提升信息化整体水平。

　　六是着力推进和谐社会建设。坚持以人为本，按照民主法治、公平正义、诚信友爱、充满活力、安定有序、人与自然和谐相处的要求，更加注重经济社会协调发展，更加注重社会公平，更加注重民主法制建设，更加注重解决关系人民群众切身利益的实际问题，深化政府管理体制改革，加强利益协调，加大矛盾纠纷调处力度，完善社会管理体系，努力构建和谐阳江。

　　　　　　　　　　　2006年工作总体要求和目标任务

　　2006年是实施“十一五”规划的第一年，做好今年的各项工作，对于我市实现“十一五”规划的奋斗目标，追赶全省发展步伐，意义十分重大。今年市政府工作的总体要求是：坚持以邓小平理论和“三个代表”重要思想为指导，全面落实科学发展观，认真贯彻党的十六大、十六届五中全会精神和省委、市委的工作部署，狠抓发展第一要务，实施“工业立市、旅游强市、农业稳市、商贸旺市、科教兴市”五大战略，以工业为主导，以招商引资为突破口，以增强自主创新能力为着力点，以优化环境为保障，加快发展临港工业和区域特色产业。坚持以人为本，构建和谐阳江。

　　今年我市经济社会发展的主要预期目标是：生产总值增长12.5%，单位生产总值能耗下降3 %，工业总产值增长17%，农业总产值增长5.5%，社会消费品零售总额增长14%，固定资产投资增长16%，外贸出口增长10%，实际利用外商直接投资增长30%，居民消费价格总水平涨幅控制在3%左右，地方财政一般预算收入增长10%，城镇登记失业率控制在3.5%以内，人口自然增长率控制在7‰以下，在岗职工年均工资实际增长6%，农村居民人均纯收入实际增长5%。

　　为实现上述目标，必须着重做好以下工作：

　　一、以工业为主攻方向，加快推进新型工业化

　　大力发展能源产业。加强与投资方的协调和配合，化解各种矛盾和纠纷，加快阳江核电站、阳西燃煤电厂、阳江抽水蓄能电站、海陵岛风力发电场等电力项目建设。充分发挥电力项目的带动作用，集聚发展上下游产业，培育产业集聚带。积极推进小水电的开发利用，力争国家和省在我市布点建设新能源项目。

　　大力发展临港工业。以省打造沿海石化产业带为契机，积极接受茂名、珠海、惠州等石化基地的产业辐射，创造条件引进若干个石化及其中下游产品加工等项目，发展装备制造、电子信息、海洋生物工程、汽车配件、钢铁等行业，培育一批新的支柱产业。重点抓好200万吨重油加工、林浆板纸一体化、德恩富光盘、特种钢、玻璃厂、龙尚鞋业等项目的跟踪落实，扶持广东丰源粮油集团、海洋石油阳江实业有限公司等企业增资扩产。

　　大力提升传统优势产业。积极运用高新技术和先进适用技术，改造提升五金刀剪、食品加工、建材机电、医药化工、家具编织、汽车配件等传统优势产业，引导五金刀剪行业加强技术创新，提高产品的科技含量和附加值，切实转变贴牌加工生产的经营模式，创立自主品牌，提高企业经营效益。着力扶持阳江宝马利有限公司、阳春轴承股份有限公司等拥有优势产品、市场前景好、带动力强、发展潜力大的企业做大做强。帮助拥有著名品牌的困难企业深化内部改革，加强经营管理，尽快走出困境，重焕生机。

　　大力发展园区工业。加强高新区和各县（市、区）工业园区的规划、建设和管理，清理闲置土地，完善配套设施，提高园区的承载力和吸引力。落实工业项目建设用地控制指标，提高单位土地投资强度和产出率。完善中山火炬（阳西）、中山石岐（阳江）两个产业转移工业园的基础设施建设，鼓励和帮助江城、阳春、阳东与珠三角等发达地区共建产业转移园区，积极承接发展一批高新技术产业，形成新的产业集群。

　　二、突出抓好招商引资，借助外力加快发展

　　落实招商引资责任制。进一步加强对招商引资工作的组织领导，严格执行《阳江市招商引资和发展工业目标考核奖惩办法（试行）》，把招商引资实绩作为考核评价干部的一项重要内容。认真落实招商引资责任，层层分解招商引资任务，调动广大干部职工招商引资的积极性，形成全民招商的新格局。

　　努力改善投资环境。严格执行市委、市政府出台的各项优惠政策，在土地、厂租、供水、供电、融资等方面调整和出台新的优惠政策，进一步降低投资门槛。简化投资审批手续，健全投资服务体系，提高办事效率和服务水平。加强口岸建设与管理，提高整体通关效能。继续抓好清费减负工作，严肃查处向企业乱收费、乱摊派、乱拉赞助等损害企业利益行为，维护企业合法利益。

　　强化招商引资措施。抢抓新一轮产业转移的良机，加强与珠三角等发达地区政府机构、外资企业协会和专业招商机构的合作，积极承接一批产业转移项目。创新招商方式，积极探索市场运作办法，在外设立招商工作站、聘请招商顾问，实行跨地区招商、驻点招商和委托招商。加强与世界500强、国内100强企业和上市公司的联系，有针对性地做好大企业大项目的引进工作。重视对欧、美、日、韩等国家和港澳台地区的招商，加大高新技术产业、装备制造业和现代服务业的招商引资力度。加强对签约项目的跟踪落实和协调服务，帮助解决项目建设中的实际问题，力促项目早入资、早上马、早出效益。

　　积极拓展国际国内市场。深化外贸体制改革，赋予更多符合条件的民营企业进出口经营权，支持中小企业和民营企业扩大出口。优化出口商品结构，扩大具有自主知识产权和自有品牌产品出口。推动出口市场多元化，巩固提高港澳、东南亚、欧美日等传统市场，大力开拓非洲、南美、印度等新市场。继续采取“政府搭台，企业唱戏”等形式，举办各种展销会、洽谈会和订货会，开展多种形式的促销活动。鼓励企业到市外办展销点、商场，建立新的销售网络，拓展我市名优特产品销售渠道，全方位开拓市内外市场，提高我市产品的市场占有率和覆盖面，推动内源型经济发展。

　　三、全面发展农业经济，建设社会主义新农村

　　大力发展现代农业。在保证粮食安全的前提下，加大农业结构调整力度，大力发展集约农业、规模农业、效益农业和特色农业，扩大具有亚热带特色的果、菜、花、茶、药、蚕桑等优势作物种植面积，力争粮经比例达40∶60。加强农科队伍建设，积极推广农业新品种、新技术，提高农业效益。大力发展特色畜牧业，推广瘦肉型生猪、黄鬃鹅等优质农畜品种繁育和养殖。加快发展生态公益林和商品用材林。

　　推进农业产业化进程。加大农业龙头企业扶持力度，培育发展一批农产品加工骨干企业，提高农产品加工水平。鼓励建立行业协会、商会等专业合作经济组织，提高农业专业化、社会化程度。抓好农业现代化示范区建设，积极应用农业先进机械和设施，加快农业现代化进程。建设完善一批农产品专业批发市场，发展农产品现代流通方式，拓宽农产品流通渠道。

　　深化农村各项改革。继续深化农村税费改革，切实减轻农民负担。加快征地制度改革，健全对被征地农民的合理补偿机制。巩固和完善农产品最低收购价制度，切实保障农民务农收益。不断优化农民进城务工经商的政策和体制环境，推进农村富余劳动力转移，发展农村劳务经济，增加农民非农收入。

　　建设社会主义新农村。按照“生产发展、生活宽裕、乡风文明、村容整洁、管理民主”的要求，全面推进农村经济、文化和社会等各项建设。坚持多予、少取、放活和工业反哺农业、城市支持农村的方针，大力发展农村经济，切实提高农民生活水平，使新农村建设带给农民实惠、受到农民拥护。加强农村规划建设和管理，积极配合省开展阳东县北惯镇平地村等村镇规划试点工作。推进农村改水、改厕、改路和住房改造等工作，解决农村 “脏、乱、差”问题，积极推广农村应用沼气，改善农村生态环境。综合治理山、水、田、林、路，加强基本农田灌区改造工程建设，改善农村耕作条件。加强农村医疗服务和公共卫生建设，推进新型农村合作医疗，改善农村疾病预防和医疗救治条件。积极引导农民移风易俗，倡导科学、文明、健康的生活方式，提高农村文明程度。

　　四、做优区域特色产业，壮大县域经济

　　加快发展民营经济。突出抓好企业技术创新平台和投资服务、人力资源服务等体系建设，发挥民营企业投诉中心作用，着力解决制约民营企业发展的突出问题。积极推进银企交流与合作，完善企业信用担保体系建设，拓宽民营经济融资渠道。鼓励和引导民营企业开展制度、技术、管理创新，提高企业自身素质，逐步发展一批主业突出、市场竞争力强的企业集团。

　　加快发展旅游业。加快旅游资源的开发利用，完善旅游配套设施，重点抓好海陵三山岛国际旅游度假区、利福泰发展有限公司五星级酒店、阳江涛景度假村等项目的建设，增强旅游服务能力，提升旅游品位。挖掘阳江文化底蕴，找准切入点，促进阳江民俗文化与旅游的融合，丰富旅游内涵。努力开发一批具有地方特色和文化内涵的旅游商品、纪念品，提高旅游收入。办好第六届旅游文化节，加大旅游宣传促销力度，拓展客源市场。积极扩大与泛珠三角的区域合作，推动区域旅游联动发展。

　　加快发展海洋产业。调整优化海洋捕捞业，突出抓好深海作业。培育沿海养殖产业带，重点加强对虾、鳗鱼等无公害养殖示范基地建设。加快发展水产品加工业，大力扶持日本川口水产有限公司、浙江舟山兴业有限公司等水产品加工项目建设和谊林海达等企业的增资扩产。加强渔业基础设施建设，重点加快闸坡国家级中心渔港和沙扒国家一级渔港建设步伐，推进东平、溪头等渔港创建国家级中心渔港、国家一级和省区域性渔港。继续跟踪落实好联合国开发署南中国海海岸带综合管理示范项目建设。办好第四届南海（阳江）开渔节，做大海洋渔业品牌。大力发展海洋生物化工、滨海旅游和海洋运输业。

　　加快发展中药生产加工业。进一步调整中药材的种植结构，扩大春砂仁、沙姜、穿心莲、板兰根、霍香、金钱草等优势品种的种植面积，努力建设一批特色明显、品质优良的中药材生产基地。构建面向农户、依托省内外各大药材公司的销售网络，确保中药材的销售畅通，促进中药材生产规模化、产业化。扶持一片天医药集团、阳江制药厂和粤西绿色工业有限公司等企业做大做强。积极引进外来资金和企业投资我市中药材加工业，提高中药材的加工能力。鼓励和支持制药企业与高等院校、科研院所开展产学研合作，加强科研攻关，创建一批中药名牌产品，提高中药材加工水平和综合效益。

　　五、加大投资力度，完善基础设施建设

　　拓宽筹资渠道。放开投资领域，鼓励社会资本以独资、合资、合作、联营、特许经营、项目融资等方式，参与市政、环保、公路、港口等项目的建设和经营。加强与银行的沟通和协调，拓展金融信贷市场，努力争取信贷资金投入基础设施建设。精心组织筛选一批重点项目，力争纳入国家、省的计划，争取上级的资金支持。

　　加快“五网”建设。加快路网建设，重点抓好阳江至云浮高速公路、S113线阳春南段二期、S369线春湾至罗定段、阳茂高速公路塘口出口至织连络线以及阳阳铁路阳江港码头专用线建设，基本实现镇通行政村公路硬底化。加快阳江港3.5万吨级通用码头建设，规划建设西部沿海高速公路连络线至程村公路和西部沿海高速公路三山出口至阳江温泉段公路。加快电网建设，着力抓好500千伏蝶岭站、220千伏阳东站、110千伏海陵站等输变电工程的前期工作，努力完成220千伏漠南站、110千伏随垌站和220千伏春城站扩容等输变电工程建设。加快气网建设，逐步完善市区管道天然气管网。加快水网建设，积极推进市自来水厂三期工程和15个镇农村饮水安全工程建设，改善城乡饮水环境。加快信息网建设，建立市级电子政务统一平台，推广公文电子交换系统。

　　加强城镇建设。认真抓好市区总体规划修编工作，编制城市近期发展规划，突出抓好城南新区、核电科研后勤基地和市区“一江两岸”的开发建设，加强城市规划区内私人住宅建设管理。加快市区高凉路、文明路、马曹路、江台公路等市政道路和城市防洪工程建设。积极开展国家卫生城市、园林城市和环保模范城市创建工作，认真抓好金山森林公园、鸳鸯湖公园改造和市区绿化建设，进一步净化绿化亮化美化城市。完成阳江市区域城镇体系规划编制工作，推进各县城和中心镇建设，增强各县城和中心镇的集聚能力。

　　六、大力发展商贸物流业，提升第三产业发展水平

　　做旺现代商贸业。充分利用区位优势和本地物产资源，加快发展商贸业。科学规划商业网点，重点发展城市购物中心，合理建设一批大型百货店。大力发展连锁店、加盟经营和授权经营，培育连锁经营品牌。加快商业企业兼并重组步伐，整合提升餐饮、娱乐等传统服务业。积极发展商务、会展等新型服务业，提升第三产业发展水平。

加快建设物流中心和各类专业市场。加快阳江港保税物流中心的建设，推进现代物流业的发展。进一步完善五金刀剪、建筑装饰材料、汽配等专业特色批发市场建设，加快第一市场改造和粤西粮油批发中心、阳江烟草配送中心建设，抓好国家棉花、粮油储备基地建设项目的跟踪落实。

　　大力发展交通运输业。以提高阳江港的通航和吞吐能力为重点，进一步完善水路、公路、铁路等交通运输体系建设，大力发展集装箱运输和石油化工、煤炭燃料的中转运输。加快交通运输企业改制和运输结构调整步伐，促进交通运输业规模化、集约化发展。

　　七、深化体制改革，增强发展活力

　　稳步推进国有企业改革。继续深化国有企业产权制度改革，加快劣势企业退出步伐。鼓励民资外资参与国有企业产权制度改革，大力发展以股份制经济为主要形式的混合所有制经济。完善国有资产监管体制，建立科学的考核分配、出资人财务监督、总会计师和法律顾问制度，确保国有资产监管到位。

　　深化投融资体制改革。加快建立市场引导投资、企业自主决策、银行独立审贷、融资方式多样、中介服务规范、宏观调控有效的新型投融资体制。鼓励金融机构创新开展以民营企业、个体户为主要服务对象的金融业务，适应民营经济的发展要求。深化农村信用社改革，大力发展现代农村金融。

　　健全财税管理机制。完善税源管理和监控体系，理顺跨县区企业税收征管关系，建立统一完整的公共收入体系。依法加强税收征管，打击偷逃骗税行为，堵塞漏洞，确保财税增长与经济增长良性互动、协调发展。继续加强“收支两条线”管理，稳妥推进国库集中收付制度改革。建立健全财政支出的有效监控机制，提高财政资金使用效益。规范政府采购行为，加强重大项目的财务监督。

　　健全现代市场体系。规范有形建筑市场和有形土地市场行为，推进产权交易市场建设。大力发展技术、劳动力等要素市场和保险市场。培育发展各类社会服务组织、行业协会（商会），健全社会服务体系。深入开展市场经济秩序专项整治，坚决打击欺行霸市、强买强卖、逃废债务等不法行为。加强诚信体系建设，逐步建立完善的信用备案制度和信用监管体系，对信用良好的企业在办照、年检、信息咨询、产品抽检和税收等方面开通“绿色通道”。

　　八、加快文化名城建设，发展社会各项事业

　　优先发展教育事业。继续深化教育体制改革，建立健全教育投入稳定增长机制，积极鼓励和引导社会力量、民间资金兴办各类教育。积极推进婴幼儿教育的发展，把婴幼儿教育纳入大教育体系。巩固提高普及九年义务教育，着力增加一批初中学位，解决班额过大和初中生流失问题；突出抓好农村基础教育，认真落实好省的部署，从今年秋季起免除所有农村户籍学生义务教育阶段杂费。大力发展普通高中教育，努力实现普通高中招生增长20%。加快推进阳江实验中学、阳春一中、阳西奋兴中学、阳东广雅中学等学校扩建、迁建和新建工作；抓好阳春二中、阳东一中、阳西一中、漠南中学等普通中学的扩招工作；加快阳江一中、阳春一中创建全国1000所示范性普通高中进程。积极发展中等职业技术教育，抓好市技工学校建设，重点培养与阳江工业发展相适应的各类专业人才，努力实现中等职业教育增长15%以上。办好高等教育，加强阳江职业技术学院基础设施建设，完善办学基本条件，提高人才培养水平。加快教育信息化建设与运用，实现优质教育资源的共享。加强教师队伍和师德师风建设，全面深入推进素质教育，提高教育质量和水平。

　　提高科技自主创新能力。鼓励扶持有条件的企业创建省级或国家级工程技术研究开发中心，建立完善以企业为主体的技术创新体系。完善市五金刀剪技术创新中心建设，为五金刀剪行业创新能力和技术水平的提升提供科技支撑。落实省市联动计划，推进专业镇建设，培育壮大专业镇产业集群。加强各类科技园区建设，大力推广应用高新技术和先进适用技术，培育发展高新技术产业。充分发挥华南理工大学阳江工业技术研究院的作用，推动企业与高校、科研院所的技术交流与合作。深化科技体制改革，完善科技服务体系。以创建省知识产权试点城市为契机，切实加强知识产权工作。加强全民科普教育，推进国民经济和社会信息化，拓展农村和社会发展领域的科技创新和服务工作。

　　发展文化事业和文化产业。以建设文化名城为目标，认真做好历史文化资源的保护和开发利用工作，加快广东海上丝绸之路博物馆建设，推进“南海一号”沉船打捞。进一步加快市图书馆、博物馆、科技馆和群众艺术馆等文化设施建设步伐，完善基层文化设施建设，巩固农村电视村村通成果，加快发展数字电视。广泛开展群众性文化活动，鼓励文艺创作，繁荣文学艺术。积极发展文化产业，培育以传媒、文博会展、电子音像、文化旅游、印刷业为支柱的文化产业体系。强化文化市场监管，全面清查取缔黑网吧、无证照电子游戏机室。加强档案馆舍和档案信息化建设，实现已公开的现行文件网上查阅。

　　促进人才、卫生、体育等社会各项事业发展。高度重视招才引智工作，加快粤西区域性人才市场建设，健全人才管理机制，构建人才服务平台，推动区域人才交流与合作，吸引优秀人才到阳江干事创业。加快疾病预防控制、卫生监督和医疗救助等公共卫生三大体系建设，积极筹建120急救指挥中心，进一步提高疾病预防控制和应对突发公共卫生事件的能力。深化医疗卫生体制改革，鼓励发展民营医院，完善社区卫生服务网络，积极推进新型合作医疗制度。整顿药品生产和流通秩序，规范医疗机构药品和医疗设备招标采购管理。大力发展中医事业，组建新阳江市中医院。深入开展爱国卫生运动，抓好对重大传染病的预防控制。深入开展艾滋病防治工作，抓好阳东县艾滋病防治试点，大力推广美沙酮替代治疗等干预和综合预防措施，坚决遏制艾滋病蔓延势头。依法加强职业病防治。深入开展全民健身运动，提高体育竞技水平，发展体育产业，加强群众性健身场所和体育设施建设。落实人口与计划生育目标责任制，完善计划生育利益导向机制，综合治理出生人口性别比偏高问题，稳定人口低生育水平，提高出生人口素质。加强全民国防教育、国民动员和民兵预备役工作，深入开展创建全国“双拥”模范城和军民共建活动。做好民政工作，巩固和深化殡葬改革。加强人民防空和气象、地震监测及预报工作。关心妇女、儿童、残疾人事业，继续做好民族、宗教、外事、侨务、文史等工作。

　　九、坚持以人为本，构建和谐阳江

　　妥善解决困难群众生产生活问题。加快实施“十项民心工程”。加大对低收入弱势群体的扶持力度，鼓励发展各类慈善团体和救助基金，健全社会救助体系。进一步完善优抚保障制度，提高优抚保障水平，积极解决优抚对象、伤残军人生活保障问题。推动老区经济发展，增加老区群众收入。继续抓好清理拖欠农民征地补偿款和建设领域拖欠工程款工作，着力解决群众反映强烈的教育、医疗、物业管理、垄断行业乱收费等问题。

　　加强就业和再就业工作。实施积极就业政策，完善公共就业服务体系。全面落实小额担保贷款、岗位补贴、社会保险补贴等再就业扶持政策，完善“零就业”家庭就业援助制度和农村就业扶贫行动，促进下岗失业人员等困难群体再就业和农村富余劳动力转移就业。加强职业技能培训，缓解就业结构性矛盾。重视做好复员转业军人安置、高校毕业生和城镇新增劳动力就业工作。

　　努力提高社会保障水平。巩固和完善社会保险市级统筹，扩大社会保险覆盖面。不断完善养老、失业、医疗、工伤、生育保险，建立健全多层次的社会保障体系。积极推进农村社会保险工作，探索被征地农民养老保险办法，健全农村安老养老机制。强化社保基金征缴和监管，提高保障水平。加强城乡居民最低生活保障的动态管理，确保符合保障标准的城乡居民应保尽保。

　　建设节约型和环境友好型社会。坚持开发节约并重，大力推进节能节水节材工作，全面推行清洁生产和绿色消费。认真做好全市土地利用总体规划修编工作，依法节约集约用地。加强环境综合治理，继续实施“青山、碧水、蓝天、绿地”工程和治污保洁工程，抓好水污染和大气污染的防治。重点抓好市第一净水厂扩建工程和各县（市、区）污水处理厂建设，加强漠阳江流域、鸳鸯湖等水源保护，加大机动车辆尾气整治力度。积极开展创建绿色城镇、绿色学校、绿色企业、绿色社区和生态示范区等活动，在全社会树立节约意识和环保意识，使节约集约资源和环境保护成为全社会的自觉行动。

　　切实维护社会稳定。积极推进社会治安防控体系建设，加大对“双抢”、“两盗”以及严重暴力和黑恶势力犯罪的打击力度，保障群众生命财产安全。突出抓好禁毒工作，严厉打击涉毒违法犯罪活动。扫除“黄赌毒”、“六合彩”赌博等社会丑恶现象。加强出租屋和流动人口管理，推进安全文明社区建设。排查和化解不稳定因素，维护社会稳定。完善创新信访机制，规范信访秩序。认真落实安全生产责任制、监督执法责任制和责任追究制，建立健全防范化解重特大安全事故长效机制。深入开展安全生产专项整治，强化执法检查监督，提高安全生产监管水平。

　　加强民主法制和精神文明建设。坚持向人大及其常委会报告工作和向人民政协通报情况制度，自觉接受监督。认真落实人大及其常委会的决议、决定，积极支持人民政协履行政治协商、民主监督、参政议政职能。广泛听取民主党派、工商联、无党派人士和人民团体意见，认真办理人大代表、政协委员的议案、建议和提案。支持工会、共青团、妇联等群众组织发挥桥梁纽带作用。健全村务公开和民主管理制度，完善村（居）民的自治机制，加强基层民主建设。加强法律宣传教育，扎实开展“五五”普法工作。深入开展“爱国、守法、诚信、知礼”现代公民教育活动，加强社会公德、职业道德和家庭美德教育，提高广大市民的道德法律素质。重视青少年思想道德建设。广泛开展文明城市、文明村镇、文明单位等群众性精神文明创建活动。

　　十、加强政府自身建设，提高行政能力和管理水平

　　建设服务政府。继续深化行政管理体制改革，进一步转变政府职能，在继续改进经济调节和市场监管方式、加强社会管理的同时，更加注重履行政府的公共服务职能，将政府的财力、物力更多地向社会发展和公共服务方面倾斜。进一步完善各级行政服务中心建设，扩大服务功能，改进服务手段，为投资者提供公开公平、优质高效的服务平台。切实加强机关作风建设，强化干部职工的服务意识，倡导特事特办、急事急办、雷厉风行的工作作风，提高政府服务水平。

　　建设务实政府。树立正确的政绩观，坚决克服官僚主义、形式主义以及铺张浪费、虚夸浮躁、急功近利行为，发扬求真务实作风，深入调查研究，做到察实情、说实话、办实事、求实效。进一步压减和控制各种会议和文件，不断改进会风和文风。健全抓落实的工作机制，强化检查监督，做到工作有部署、有检查、有落实。重点加强对市委、市政府各项重大决策、重点工作落实情况的督查，推动各项工作落到实处，取得实效。

　　建设法治政府。完善政府决策机制，规范决策程序。切实加强法制教育，提高法律素质，增强法制观念。继续贯彻落实《行政许可法》和《全面推进依法行政实施纲要》，提高各级政府依法决策、依法办事能力。运用法律手段管理经济和社会事务，提高法制化管理水平。推行行政执法责任制，加强执法监督，维护司法公正，使依法行政落实到政府行为的各个方面、各个环节。

　　建设廉洁政府。以实施《公务员法》为契机，进一步规范公务员从政行为，严格按照法定权限和程序行使权力、履行职责。落实廉政建设责任制，加强党风廉政建设。坚持标本兼治、综合治理，建立完善教育、制度、监督并重的惩治和预防腐败体系，坚决纠正损害群众利益的行为，严肃查处违纪违法的大案要案，努力建设一支政治坚定、业务精通、清正廉洁、作风优良的公务员队伍。

　　各位代表，今年是实施“十一五”规划的开局之年，责任重大，使命光荣。让我们高举邓小平理论和“三个代表”重要思想伟大旗帜，紧密团结在以胡锦涛同志为总书记的党中央周围，全面落实科学发展观，按照省委、省政府和市委的工作部署，进一步解放思想，抢抓机遇，开拓进取，扎实工作，为实现“十一五”规划开好头、起好步，为开创我市社会主义现代化建设新局面而努力奋斗！
